# Determinants of neural tube defects among newborns in public referral hospitals in Eastern Ethiopia

**DOI:** 10.1186/s40795-023-00752-7

**Published:** 2023-07-25

**Authors:** Fadumo Ahmed Mohamed, Merga Dheresa, Temam Bashir Raru, Newas yusuf, Tahir Ahmed Hassen, Ame Mehadi, Tara Wilfong, Kedir Negesso Tukeni, Mohammed Abdurke Kure, Kedir Teji Roba

**Affiliations:** 1 School of Public Health, College of Health and Medical Sciences, Haramaya University, Harar, Ethiopia; 2 School of Nursing and Midwifery, College of Health and Medical Sciences, Haramaya University, P.O.BOX: 235, Harar, Ethiopia; 3 Department of Internal Medicine, Jimma University, Jimma, Ethiopia

**Keywords:** Neural tube defect, Determinants, Maternal, Newborn, Eastern Ethiopia

## Abstract

**Background:**

Neural tube defects (NTDs) are serious brain and spine birth defects. Although NTDs are primarily pregnancy complications, such as abortion and stillbirth, they also contribute to under-five morbidity and mortality, as well as long-term disability and psychological impact. Despite these negative outcomes, the determinants of NTDs are not widely studied in Ethiopia, particularly in the country’s east. As a result, we sought to identify the risk factors for NTDs in neonates born in public referral hospitals in eastern Ethiopia.

**Methods:**

A facility-based unmatched case-control study was carried out at Hiwot Fana Comprehensive Specialized Hospital and Sheik Hassen Yabare Jigjiga University Referral Hospital in Eastern Ethiopia. We included 59 cases identified in the selected facilities between September 10, 2021, and February 5, 2022, and 118 control neonates, with a case-to-control ratio of 1:2. Data were gathered through the use of interviewer-administered questionnaires and medical record review. To identify determinant factors of NTDs, a multivariable logistic regression model was used, which included all predictor variables from the bivariable analysis. The results were reported using an Adjusted Odds Ratio (AOR) with a 95% confidence interval. A p-value of < 0.05 was considered statistically significant.

**Results:**

In total, 59 cases of NTDs were identified out of 2915 live birth total births registered in the two hospitals, making the incidence of NTDs 202.4/10,000 births. In the final model analysis, determinant factors such as gender of newborn [AOR = 2.97; 95%CI(1.27, 6.92)], having no history of antenatal care[AOR = 4.45;95%CI(1.30,15.20)], having a poor food consumption score (AOR = 3.38;95% CI;1.06,10.72), having history of monotonous diet consumption (AOR = 4.80; 95%CI: 1.09, 9.08; P = 0.038), and coffee consumption of three or more cups per day during pregnancy (AOR = 3.84:95% CI: 1.23, 11.97) were statistically associated with NTDs.

**Conclusion:**

Modifiable and non-modifiable determinants were identified as major contributors of neural tube defect in this study. Early screening, dietary intervention counseling to increase consumption of a healthy diet, coffee consumption reduction, and pre-pregnancy supplementation programs should be developed to reduce NTDs in Ethiopia.

## Introduction

Neural tube defects (NTDs) are complex congenital anomalies caused by neural tube malformations that affect the developing embryo’s brain and/or spine during the first eight weeks of embryogenesis. Primary neurulation occurs between 20 and 28 days of gestation and results in anencephaly, myelomeningocele (open spina bifida), and craniorachischisis, whereas secondary neurulation occurs between 45 and 53 days of gestation and results in spina bifida occulta to severe spinal cord tethering [1–5].

Although the exact causes of NTDs are unknown, evidence points to a wide range of possible causes [6–8]. According to the multifactorial threshold model, the majority of NTDs are caused by the additive interaction of risk factors such as socioeconomic status, micronutrient deficiency, and maternal health and genetic issues such as valproic acid ingestion [6, 8, 9]. Since embryonic, development is determined by the maternal body composition, size and total nutrient store, the maternal nutritional status influences pregnancy outcome and is linked to NTDs [10]. Reduced folic acid intake during preconception and early pregnancy raises the risk of having a fetus with NTDs [1].

Every year, more than 300,000 newborns worldwide are diagnosed with NTDs [2, 3]. Furthermore, NTDs are responsible for 88,000 annual deaths and 8.6 million disability-adjusted life years (DALYs) worldwide [2, 3]. Every year, approximately 190,000 newborns in low- and middle-income countries (LMICs) are affected by NTDs [4]. Although the prevalence of NTDs has decreased in developed countries, it remains four times higher in developing countries [5]. An estimated 260,100 NTD cases, equivalent to 1.9 per 1000 live births, were reported globally in 2015 alone [5, 6]. In Africa, however, the risk of NTDs ranges from 1 to 11 per 1000 births per year [5, 7–12]. For example, in a Tanzanian study, NTDs were the most common congenital anomalies among significant external structural defects, affecting 1 in 1000 births [11]. NTDs are the second leading cause of birth defects in Ethiopia, where the prevalence of congenital disabilities reaches 19 per 1000 births [2, 6, 13–15]. Different studies found varying rates of NTDs. In Ethiopia, the overall incidence of NTDs exceeds 13 per 1000 births [16]. The most recent hospital-based studies in Tigray and Addis Abeba show that Ethiopia has a very high incidence rate of NTDs. The overall occurrence of NTDs in Tigray was 13.1 per 1,000 births, but when broken down by zone, it was 30.4 in Southern Tigray and 89 in Central Tigray [17].

The rate is ten times higher than the report from eight WHO member countries in Africa (1.0 to 2.0 per 1,000 live births) and 26 times higher than it should be [17]. A study conducted in two teaching hospitals in Addis Abeba, Ethiopia, revealed an overall incidence of 6.1 per 1000 births [12]. Furthermore, 48.7% of pregnancy terminations in Ethiopia after 12 weeks were reported to be due to NTDs [15]. The 2016 prevalence of spina bifida and anencephaly in Ethiopia was estimated to be 13 per 1,000 total births, with a prevention goal of 0.5 per 1,000 total births [16].

Low socioeconomic status, maternal exposure to environmental factors (e.g., pesticides and chemicals), maternal smoking during pregnancy, genetic factors, delaying childbearing, low folic-acid supplementation before or during pregnancy, fetal sex, and lack of antenatal care have all been linked to NTDs [15, 18–21]. Furthermore, evidence suggests that maternal exposure to valproic acid, an anti-epileptic and mood-stabilizing drug, during pregnancy increases the risk of having a fetus affected with NTDs [2, 22, 23].

Several studies have looked into the relationship between maternal caffeine consumption and birth defects. Caffeine use by mothers has been linked to an increase in the risk of neonatal birth defects ranging from 1.3 to 1. 0.8 [24] The study was carried out at Debre Berhan Specialized Hospital in North Eastern Ethiopia [25]. Over the last decade, Ethiopia has made significant strides in combating malnutrition. The Ethiopian government launched the National Nutrition Program (NNP) in 2008, gradually expanding its scope. The National Food and Nutrition Policy (NFNP) promotes a coordinated and comprehensive approach to food and nutrition security, emphasizing the importance of access to nutritious foods [26–29]. Furthermore, the Sequota Declaration Implementation Plan aims to end undernutrition by 2030 and has encouraged collaboration across sectors [26, 28–31].

Despite the government’s efforts to implement a variety of interventions to address the issue of dietary diversity, many Ethiopians’ diets remain monotonous, with little variation in diet diversity. Despite rapid changes in food systems in Ethiopia, dietary diversity remains a challenge [28, 29]. Similarly, the Ethiopian government has supported and implemented a number of high-impact nutrition interventions to improve the nutritional status of the population, particularly the most vulnerable groups (women and children). These include monthly nutrition counseling, antenatal care visits, post-natal care, increasing the production of nutritious foods, improving household dietary diversity, and fortifying grains with iron and folic acid [28, 29].

Folate deficiency and insufficiency are widespread public health issues in Ethiopia. Based on red blood cell folate levels, a recent assessment based on the most recent national micronutrient survey discovered that 84% of women of reproductive age are at risk of having a child with NTDs [15]. NTD research is uncommon in Ethiopia, particularly in the eastern region. The burden of NTDs was 5.71% among neonates admitted to the neonatal intensive care unit at Hiwot Fana Specialized Hospital (HFCSH) in Harar in a cross-sectional study of NTDs and associated factors [32]. A retrospective cohort study of neonates born in three referral hospitals in Ethiopia revealed an unacceptably high rate of NTDs, 107.5 per 10,000 live births, which was five times higher than the WHO estimate for Ethiopia [10]. Several studies suggested that case-control or cohort studies be used to identify the main determinants of NTDs and reduce the burden. As a result, the purpose of this study was to identify the risk factors for NTDs in neonates born in public referral hospitals in Eastern Ethiopia.

## Methods and materials

### Study setting and period

The study was conducted among neonates born in two public referral hospitals in Eastern Ethiopia, Hiwot Fana Comprehensive Specialized Hospital (HFCSH) and Sheik Hassen Yabare Jigjiga University Referral Hospital (SHJJURH), in Harar and Jigjiga town, respectively, from September 10, 2021 to February 5, 2022. HFCSH, one of Harar’s oldest hospitals, provides comprehensive referral services to the entire population of the country’s eastern region, with an average of 11,957 admissions and 5808 deliveries per year [33–35].

SHJJURH is Jigjiga University’s teaching center and the busiest referral hospital in the Somali Regional State. The hospital serves a population of over seven million people living in all zones and districts of the Somali Region State, neighboring districts in the eastern part of Oromia, and a large portion of neighboring Somalia. Every year, the hospital serves over 38,523 outpatients, 7,690 hospitalized patients, 3,434 deliveries, and 9,270 emergency cases [36].

### Population and study design

A facility-based unmatched case-control study was conducted at HFCSH and SHJJURH from September 10, 2021, to February 5, 2022. In this study, the cases were all neonates born after 28 weeks of gestation (the age of viability for developing countries) with a confirmed diagnosis of any form of NTDs, while the controls were all neonates born after 28 weeks of gestation with no type of NTDs, no other anomalies, and no other anomalies. Pregnancies that were terminated before 28 weeks, as well as neonates whose mothers were unavailable or critically ill, were excluded from both the case and control groups.

### Sample size determination and sampling techniques

The sample size for this study was determined by taking into account the various factors that contribute to NTDs. As a result, the sample size was calculated using Epi-Info statistical software (version 7.1.0) with the following assumptions: the proportion of cases with a history of abortion (18.7%) and the proportion of exposed controls (4.8%) with a 4.9 odds ratio from similar studies [14, 37]. a and a case-to-control ratio of 1:2 to achieve 80% power at 95% CI, with a 5% contingency for non-responses, the final sample size was 180. (60 cases and 120 controls). However, this study included 59 cases and 118 controls who met the inclusion criteria. This study’s participants were chosen using consecutive and nonprobability convenience sampling techniques. Two consecutive controls were chosen for each eligible case using the nonprobability convenience-sampling technique until the required final sample size was reached.

### Data collection tool and procedure

A structured questionnaire that had been utilized in Ethiopia before [32, 38, 39] was employed in this investigation. The questionnaire was prepared in English first, then translated into local languages (Afan Oromo, Amharic, and AF Somali), and then back to English by language experts to ensure consistency. Data were collected by midwives or nurses working in the labor wards of each selected hospital, and data collectors received one-day training. A chart review was performed to confirm the diagnosis and to obtain baseline data. Both cases and controls were interviewed by the same interviewer. To ensure their privacy and to encourage participation, mothers were interviewed in private rooms.

### Study variables and operational definitions

The dependent variable of this study was NTDs defined based on the ICD-10 criteria [15], and the controls were neonates born without NTDs in the selected study hospitals. The independent variables were socio-demographic characteristics (age of the mother, residence area, marital status, level of education, income, and sex of the newborn), maternal lifestyle (maternal coffee consumption, maternal alcohol consumption, maternal khat chewing, and smoking habit), maternal obstetric and medical determinants (parity, ANC follow for indexed pregnancy, planned pregnancy, previous history of NTDs, medical problems during the pregnancy, previous adverse birth outcomes: stillbirth, preterm birth, low birth weight, abortion, small for gestational age), maternal medication-related factors (drug use, folic acid or multivitamin supplementation) and environmental factors (chemical exposure, radiation/heat exposure).

### Data Quality Control

Prior to data collection, a pretest was performed on 5% of the sample size at Karamara General Hospital to ensure that the questionnaire was functional, and any necessary changes were made. A one-day training was provided for data collectors and supervisors prior to data collection. Every day, all collected data was double-checked for completeness.

### Data processing and analysis

Data was collected, checked for completeness, entered into EpiData version 3.1, and then exported and analyzed with SPPS version 25. The data was examined to ensure that it was normal and that other assumptions were met. The association between each independent variable and NTDs was evaluated using binary logistic regression analysis. An adjusted odds ratio was used in multivariable analysis to identify independent determinants of NTDs. In the bivariate analysis, variables with a p-value less than 0.25 were considered candidates for the multivariable analysis. The strength of the association between the independent variables associated with NTDs was measured using an adjusted odds ratio with a 95% confidence interval, and statistical significance was defined as a P-value less than 0.05.

#### Ethical consideration

Haramaya University College of Health and Medical Science Institutional Health Research Ethics Review Committee provided ethical approval prior to data collection (IHRERC). Before data collection, We obtained consent directly from all people above the age of sixteen, regardless of their educational background. In fact, there are no legal guardians under the age of sixteen in our study. We confirm that all experiments were carried out in accordance with applicable guidelines and regulations (such as the Declaration of Helsinki). Participants were told they could refuse or withdraw at any time. To maintain confidentiality, their names and other personal identifiers were not registered. Furthermore, the interviews were conducted in a separate room to ensure confidentiality.

## Results

### Sociodemographic characteristics of the participants

This study included 59 cases and 118 controls, with a 98% response rate. When compared to the control group, a higher proportion of mothers in the case group were under the age of 25 (55.9% vs. 33.9%). When comparing maternal age at first childbirth, more mothers in the case group (86.4%) were under 25 years old than mothers in the control groups (61.9%). In comparison to their counterparts (29.7%), more than one-third (37.3%) of case mothers received only primary education. The majority of case mothers were housewives, as were their counterparts. (Table 1).

**Table 1:** Sociodemographic characteristics of participants in Eastern Ethiopia, 2022(N = 177)

Variables	Category	Cases (N = 59)	Controls(N = 118)	X^2^ (P-Value)
		n (%)	n(%)	
Maternal age (in years)	15–24	33 (55.9)	40(33.9)	0.019(0.146)
	25–34	22(37.3)	66(55.9)	
	≥ 35	4(6.8)	12(10.2)	
Maternal age at 1st childbirth	15–24	51(86.4)	73(61.9)	0.004(0.005)
	25–34	8(13.6)	45(38.1)	
Residence	Rural	21(35.6)	34(28.8)	0.358(0.359)
	Urban	38(64.4)	84(71.2)	
Marital status	Single	2(3.4)	3(2.5)	0.748(0.749)
	Married	57(96.6)	114(97.5)	
Maternal educational level	Illiterate	20(33.9)	20(16.9)	0.008(0.008)
	Primary	22(37.3)	35(29.7)	
	Secondary	14(23.7)	44(37.3)	
	Tertiary	3(5.1)	19(16.1)	
Maternal occupation	House wife	48(81.4)	75(63.6)	0.098(0.162)
	Merchant	4(6.8)	20(16.9)	
	Daily laborer	4(6.8)	11(9.3)	
	Office worker	3(5.1)	12(10.2)	
Sex of the newborn	Male	23(39)	65(55.1)	0.043(0.045)
	Female	36(61)	53(44.9)	

### Maternal obstetric, medical and newborn-related conditions

During the index pregnancy, a higher proportion of mothers in cases (30.5%) did not receive antenatal care than mothers in controls (16.5%). Furthermore, a higher proportion of case mothers (81.4%) than control mothers (73.7%) did not receive supplemental folic acid. In terms of adverse birth outcomes, such as stillbirth, preterm birth, low birth weight, and small for gestational age, similar differences were observed between mothers in the two groups (17% for cases and 11% for controls). Finally, the proportion of medical problems during pregnancy, such as diabetes, hypertension, human immunodeficiency virus, and urinary tract infections, was higher among case mothers compared to control mothers (15.3% vs. 9.3%, respectively). (Table 2).

**Table 2:** Maternal obstetric, medical and newborn-related conditions in Eastern Ethiopia, 2022(N = 177)

Variables	Category	Cases (N = 59)	Controls (N = 118)	X^2^ (p-value)
		N (%)	N (%)	
Gravidity	Prim Gravida	3 (5.1)	5 (4.2)	0.934 (0.85)
	Multigravida	33 (55.9)	69 (58.5)	
	Grand multipara	23 (39)	44 (37.3)	
Parity	Prim para	10 (17)	15 (12.7)	0.581(0.84)
	Multipara	37 (62.7)	83 (70.3)	
	Grand multipara	12 (20.3)	20 (17)	
Planned pregnancy	Yes	43 (72.9)	92 (78)	0.453(0.454)
	No	16 (27.1)	26 (22)	
ANC visits	No visits	18 (30.5)	20 (16.5)	0.008 (0.003)
	1–2 visits	33 (55.9)	58 (49.2)	
	3–4 visits	8 (13.6)	40 (33.9)	
Folic Acid supplementation	Yes	11(18.6)	31(26.3)	0.26 (0.26)
	No	48 (81.4)	87 (73.7)	
Mode of delivery	SVD	54 (91.5)	110 (93.2)	0.77 (0.48)
	C-section	3 (5.1)	6 (5.1)	
	Instrumental	2 (3.4)	2 (1.7)	
Onset of labor	Induced	5 (8.5)	8 (6.8)	0.684 (0.684)
	Spontaneous	54 (91.5)	110 (93.2)	
Previous adverse birth outcomes	Yes	10 (16.9)	13 (11)	0.26 (0.27)
	No	49 (83.1)	105 (89)	
Medical problems during pregnancy	Yes	9 (15.3)	11 (9.3)	0.24 (0.244)
	No	50 (84.7)	107 (90.7)	

### Maternal lifestyle, behavioral, and nutritional related conditions

In this study, more case mothers (32.2%) drank three or more cups of coffee per day than control mothers (15.3%). When compared to their counterparts (40.7%), more than half (55.9%) of case mothers had low food consumption scores. When compared to control mothers (33.1%), the majority of case mothers (64.4%) consumed monotonous diets. In terms of substance use during pregnancy, 13.6% of cases and 9.3% of controls used drugs, alcohol, khat, or cigarettes.**(**Table 3**).**

**Table 3:** Maternal lifestyle, behavioral and nutritional conditions in Eastern Ethiopia, 2022 (N = 177)

Variables	Category	Cases (N = 59)	Controls (N = 118)	X^2^	P-value
		N (%)	N (%)		
Daily coffee or tea consumption	No regular daily consumption	14 (23.7)	42(35.6)	0.025	0.011
	1–2 cups per day	26 (44.1)	58 (49.2)		
	3 or more cups per day	19 (32.2)	18 (15.3)		
Maternal fever	Yes	13 (22)	18 (15.3)	0.26	0.26
	No	46 (88)	100 (84.7)		
Substance use during pregnancy	Yes	8 (13.6)	11 (9.3)	0.39	0.393
	No	51(86.4)	107 (90.7)		
Passive smoking	Yes	12 (20.3)	17 (14.4)	0.31	0.317
	No	47 (79.7)	101 (85.6)		
Food consumption Score	Poor	33 (55.9)	48 (40.7)	0.05	0.021
	Border line	20 (33.9)	42 (35.6)		
	Acceptable	6 (10.2)	28 (23.7)		
Stable Food	Sorghum with watt	2 (3.4)	2 (1.7)	0.44	0.685
	Tef Injera + watt	40 (67.8)	90 (76.3)		
	Rice/Pasta	17 (28.8)	26 (22)		
Food diversity	Monotonies diet	38 (64.4)	51 (33.1)	0.011	0.008
	Sometimes different	17 (28.8)	42 (42.4)		
	Always different	4 (6.8)	25 (24.6)		
Home gardening	Yes	3 (5.1)	10 (8.5)	0.41	0.42
	No	56 (94.9)	108 (91.5)		

Key: *=Khat chewing, Alcohol, Cigarette smoking

### Incidences of NTDs

Both hospitals recorded 2915 live births during the study period. 1836 of them were from Hiwot Fana Specialized University Hospital, while the rest were from Jigjiga University Referral Hospital. During the study period, 59 cases of NTDs were identified, 39 of which were from Hiwot Fana Specialized University Hospital and 20 from Jigjiga University Referral Hospital. Thus, the incidence of NTDs was 20.24/1000 births, with Hiwot Fana Specialized University Hospital having the highest incidence at 21.24/1000 births. (Table 4).

**Table 4:** Incidence of NTD study sites

Facilities	Total live birth in past six months	NTD cases in the past six months	Incidences /10,000
Hiwot Fana	1836	39	212.4
Jigjiga University Referral Hospital	1079	20	185.4
Total	2915	59	202.4

### Determinants of NTDs

In the bivariate analysis, maternal age, maternal age at first childbirth, residence, educational status, occupation, sex of newborn, planned pregnancy, folic acid supplementation, ANC visits, previous adverse birth outcome, medical problem, maternal fever during pregnancy, daily coffee consumption, substance use, food consumption score, and dietary diversification were found to be associated with NTDs at a p-value of 0.25; thus, all of these factors were considered for multivariable logistic regression analysis.

After adjusting for potential confounders, female sex of the newborn, lack of antenatal care follow-up, poor maternal food consumption score, and coffee consumption of three or more cups per day during pregnancy were found to be significantly associated with NTDs at a p-value of 0.05 in the multivariable logistic regressions.

Female newborns had nearly three times the odds of having NTDs as males (AOR = 2.97; 95%CI: 1.27, 6.92). A child born to a mother who had no antenatal care visits was four and a half times more likely to develop an NTD than their counterparts (AOR = 4.45; 95%CI: 1.30, 15.20). Mothers with low food consumption scores were more than three times more likely to have NTDs than those with acceptable scores (AOR = 3.38;95%CI;1.06,10.72). Mothers who drank three or more cups of coffee or tea per day were four times (AOR = 3.84:95% CI: 1.23, 11.97) more likely to have a baby with NTDs than mothers who drank less than three cups per day. Maternal dietary diversification was strongly associated with the risk of developing NTDs in newborns. When compared to a more diverse diet, a mother with a monotonous diet was nearly 25 times (AOR = 24.80; 95%CI: 1.09, 21.08) more likely to have a baby with NTDs. (Table 5).

**Table 5:** Multivariable analysis of determinants of NTDs in Eastern Ethiopia, 2022(N = 177)

Variables	Category	Cases N (%)	Controls N (%)	COR (95%CI)	AOR (95%CI)
Current maternal age	15–24	33 (55.9)	40 (33.9)	2.47 (0.73–8.39)	11.19 (0.52, 2.74)
	25–34	22 (37.3)	66 (55.9)	1 (0.29–3.42)	1.57 (0.34, 7.30)
	≥ 35	4 (6.8)	12 (10.2)	Ref	
Maternal age at 1st birth	15–24	49 (83.1)	73 (61.9)	3.02 (1.39–6.55)	4.25 (1.57, 11.49)^*^
	25–34	10 (16.9)	45 (38.1)	Ref	Ref
Maternal level of education	Illiterate	20 (33.9)	20 (16.9)	6.3 (1.6–24)	7.42 (0.50, 109.19)
	Primary	22 (37.3)	35 (29.7)	3.9 (1–15)	8.37 (0.60, 116.40)
	Secondary	14 (23.7)	44 (37.3)	2 (0.51–7.8)	3.23 (0.25, 41.33)
	Tertiary	3 (5.1)	19 (16.1)	Ref	Ref
Sex of new born	Female	36 (61)	53 (44.9)	1.91(1.02–3.63	2.97 (1.27, 6.92)^*^
	Male	23 (39)	65 (55.1)	Ref	
Maternal occupation	House wife	48 (81.4)	75 (63.6)	2.56 (0.68–9.54)	0.64 (0.048, 8.72)
	Merchant	4 (6.8)	20 (16.9)	0.80 (0.15–4.2)	0.23 (0.01, 3.11)
	Daily laborer	4 (6.8)	11(9.3)	1.45 (0.26-8)	0.21 (0.01, 4.18)
	Office worker	3 (5.1)	12 (10.2)	Ref	Ref
ANC visits	No visits	18 (30.5)	20 (16.5)	4.5 (1.67-12)	4.45 (1.30, 9.20)^*^
	1–2 visits	33 (55.9)	58 (49.2)	2.8 (1.19–6.7)	3.30 (1.14, 9.58)^*^
	3–4 visits	8 (13.6)	40 (33.9)	Ref	Ref
Medical problems	No	50 (84.7)	107 (90.7)	0.57 (0.22–1.26)	0.61 (0.17,2.17)
	Yes	9 (15.3)	11 (9.3)	Ref	Ref
Coffee consumption	No regular daily consumption	14 (23.7)	42 (35.6)	Ref	Ref
	1 to 2 cups	26 (44.1)	58 (49.2)	1.34 (0.63, 2.88 )	1.31 (0.51, 3.37 )
	3 cups or more	19 (32.2)	18 (15.3)	3.17 (1.31, 7.66)	3.84 (1.23, 11.97)^*^
Food consumption score	Poor	33 (55.9)	48 (40.7)	3.2 (1.19–8.6)	3.38 (2.06, 7.72)^*^
	Border line	20 (33.9)	42 (35.6)	2.2 (0.79–6.22)	2.91(0.87, 9.65)
	Acceptable	6 (10.2)	28 (23.7)	Ref	Ref
Dietary diversity	Monotonous diet	38 (64.4)	51 (33.1)	4.65 (1.49–14.5)	4.20 (3.09, 9.08)*
	Sometimes different	17 (28.8)	42 (42.4)	2.53 (0.76–8.3)	2.57 (0.55, 11.92)
	Always different	4 (6.8)	25 (24.6)	Ref	Ref

**Key**: *p-value < 0.05; COR: Crude Odds Ratio; AOR; Adjusted Odds Ratio; CI: Confidence Interval

## Discussion

In total, 59 cases of NTDs were identified out of 2915 live birth total births registered in the two hospitals, making the incidence of NTDs 202.4/10,000 births. This study revealed that lack of antenatal care follow-up, newborn sex, coffee consumption, and poor food consumption score had an association with NTDs.

This finding indicated that inadequate antenatal care was independently associated with NTDs, which is consistent with findings from a study conducted in four referral hospitals in the Amhara region [40] and an Iranian study [41]. This could be because pregnant women who do not receive antenatal care may not receive adequate information, particularly dietary counseling, including the importance of folic acid supplementation. As a result, these women are more likely to experience pregnancy, birth, and postpartum complications [38].

This study discovered that young maternal age at first childbirth was a risk factor for NTDs, which was consistent with the findings of a case-control study conducted at Zewditu Memorial Hospital in Addis Abeba, Ethiopia. Low socioeconomic status, low educational level, and lack of knowledge about potential risk factors and preventive measures (dietary habits, access to health care) may increase the risk of NTDs during pregnancy in younger age groups [39].

In this study, poor food consumption was linked to NTDs. Another study conducted in Ethiopia’s Tigray region yielded similar results [18]. Healthy maternal dietary patterns, as measured by diet quality scores, were linked to a lower risk of NTDs and clefts. These findings suggest that nutritional interventions could reduce the risk of major birth defects even further, supplement existing efforts to fortify foods, and encourage periconceptional multivitamin use [42]. Unfortunately, the mechanism by which folic acid reduces the risk of having a child with an NTD is not completely understood [43].

In this study, being a female newborn increased the risk of NTDs threefold when compared to males. This finding is consistent with findings from three teaching hospitals in Addis Abeba, Ethiopia [15], Tigray, Ethiopia [18], and Algeria [44]. However, it is unclear how the prevalence of NTDs varies by gender [45]. As a result, no single explanation explains why NTDs affect more female newborns than males, necessitating additional research.

Coffee consumption was linked to NTDs. This finding is consistent with hospital-based studies conducted in Addis Abeba, Ethiopia [39, 46]. Although the exact mechanisms by which coffee consumption and NTD risks are not clear, several studies have shown that coffee consumption during pregnancy can cause abortion, low birth weight, and changes in folate metabolism (acting as a vitamin B6 antagonist, possibly impairing homocysteine breakdown) [47, 48] [49]. Caffeine has the potential to cross the placenta during pregnancy. Total caffeine consumption of 10 mg per day increased the risk of NTDs [49, 50]. Caffeine raises homocysteine levels, and high homocysteine levels are linked to an increased risk of NTD. This explains one possible explanation for the link between total caffeine and NTDs. Other previously evidenced probable mechanisms of caffeine include crossing the placenta during pregnancy, synergistic effects with other teratogens, inhibitory effects on DNA repair, and release of catecholamines or corticosterone [47–49]. Therefore, further studies are required to understand the exact mechanisms by which coffee consumption is associated with NTDs.

### Strength and limitations of the study

A strong study design was used in this study (case control). Despite the rarity of the cases, we collected primary data that was confirmed by a physician. Because some of the variables in this study were sensitive (for example, substance use during pregnancy), there might be social desirability bias. Second, the study was conducted in a hospital, and home births were not included. Finally, the study did not address the genetic and chromosomal determinants of NTDs, which folic acid does not prevent. Furthermore, the other limitations were due to the small sample size.

## Conclusion

According to this study, female newborns, few antenatal care visits, maternal coffee consumption of three or more cups per day during pregnancy, and a low food consumption score were all independently associated with NTDs. As a result, comprehensive preventive strategies focusing on the identified risk factors are required at the regional and national levels.

The findings suggest that primary preventative strategies, such as active promotion of preconception care services and food fortification, as well as promoting good dietary practices and household food diversification, should be strengthened in order to reduce the burden of NTDs. Furthermore, ensuring that mothers avoid harmful substances, particularly alcohol and tobacco, increasing and strengthening the education of health staff and others involved in promoting the prevention of congenital anomalies, pre-and peri-conception, and neonatal screening, developing and improving registration and surveillance systems, reducing coffee and tea consumption during pregnancy, improving diets for adolescent girls and women of reproductive age, and developing e-prevention programs are all priorities. Finally, further research is recommended to address the genetic factors of NTDs.

## Data Availability

The datasets used and/or analyzed during the current study available from the corresponding author on reasonable request.

## Abbreviations

ANC: Antenatal care
AOR: Adjusted Odds Ratio
CI: Confidence Interval
CNS: Central Nervous System
DM: Diabetes Mellitus
EDHS: Ethiopian Demographic and Health Survey
GA: Gestational age
HFCSH: Hiwot Fana Comprehensive Specialized Hospital
HTN: Hypertension
HIV: Human Immune Virus
ICD: International Classification of Disease
JJURH: Jigjiga University Referral Hospital
LBW: Low Birth Weight
LMIC: Low and Middle Income Countries
NTDs: Neural Tube Defects
OR: Odds Ratio
PROM: Premature rupture of membrane
SGA: Small for gestational age
UTI: Urinary Tract Infection
WHO: World Health Organization

## References

[1] Williams J et al. Updated estimates of neural tube defects prevented by mandatory folic acid fortification—United States, 1995–2011. MMWR. Morbidity and mortality weekly report, 2015. 64(1): p. 1. PMC4584791 25590678

[2] Flores A (2014). Global burden of neural tube defects, risk factors, and prevention. Indian J community health.

[3] Sutton M, Daly LE, Kirke PN (2008). Survival and disability in a cohort of neural tube defect births in Dublin, Ireland. Birth Defects Res A Clin Mol Teratol.

[4] Lo A, Polšek D, Sidhu S. Estimating the burden of neural tube defects in low–and middle–income countries. J Global health, 2014. 4(1). 10.7189/jogh.04.010402 PMC4073251 24976961

[5] Blencowe H (2018). Estimates of global and regional prevalence of neural tube defects for 2015: a systematic analysis. Ann N Y Acad Sci.

[6] Bitew ZW (2020). Magnitude and Associated factors of neural tube defects in Ethiopia: a systematic review and Meta-analysis. Global Pediatr health.

[7] Ibrahim Z (2016). Describing the prevalence of neural tube defects Worldwide: a Systematc Literature Review. PLoS ONE.

[8] Farkas SA (2013). Epigenetic alterations in folate transport genes in placental tissue from fetuses with neural tube defects and in leukocytes from subjects with hyperhomocysteinemia. Epigenetics.

[9] Alhassan A, Adam A, Nangkuu D (2017). Prevalence of neural tube defect and hydrocephalus in Northern Ghana. J Med Biomedical Sci.

[10] Berhane A, Belachew T (2022). Trend and burden of neural tube defects among cohort of pregnant women in Ethiopia: where are we in the prevention and what is the way forward?. PLoS ONE.

[11] Kishimba RS et al. Birth prevalence of selected external structural birth defects at four hospitals in Dar es Salaam, Tanzania, 2011–2012. J global health, 2015. 5(2). 10.7189/jogh.05.020411 PMC4562455 26361541

[12] Sorri G, Mesfin E (2015). Patterns of neural tube defects at two teaching hospitals in Addis Ababa, Ethiopia a three years retrospective study. Ethiop Med J.

[13] Kheir AEM, Eisa WMH. Neural tube defects; clinical patterns, associated risk factors and maternal awareness in Khartoum state, Sudan. 2015.

[14] Taye M (2016). Magnitude of birth defects in central and Northwest Ethiopia from 2010–2014: a descriptive retrospective study. PLoS ONE.

[15] Gedefaw A, Teklu S, Tadesse BT. Magnitude of neural tube defects and associated risk factors at three teaching hospitals in Addis Ababa, Ethiopia. BioMed research international, 2018. 2018. 10.1155/2018/4829023 PMC5866884 29713643

[16] Dixon M (2019). High potential for reducing folic acid-preventable spina bifida and anencephaly, and related stillbirth and child mortality, in Ethiopia. Birth Defects Research.

[17] Berihu BA (2018). High burden of neural tube defects in Tigray, Northern Ethiopia: hospital-based study. PLoS ONE.

[18] Berihu BA (2019). Maternal risk factors associated with neural tube defects in Tigray regional state of Ethiopia. Brain Develop.

[19] Behrooz A, Gorjizadeh MH (2007). Prevalence and correlates of neural tube defect in south West Iran: retrospective analysis. Sultan Qaboos University Medical Journal.

[20] Organization WH. Congenital anomalies, 2016. Geneva (CH): WHO, 2016.

[21] Øyen N (2019). Association between maternal folic acid supplementation and congenital heart defects in offspring in birth cohorts from Denmark and Norway. J Am Heart Association.

[22] Mohmmed RGA (2019). Determination of risk factors Associated with neural tube defects in infants. Int J Nurs.

[23] Denny KJ (2013). Neural tube defects, folate, and immune modulation. Birth Defects Research Part A: Clinical and Molecular Teratology.

[24] Browne ML (2011). Maternal caffeine intake and risk of selected birth defects in the National Birth Defects Prevention Study. Birth Defects Res A Clin Mol Teratol.

[25] Mulu GB et al. Factors Associated with neural tube defects among newborns delivered at Debre Berhan Specialized Hospital, North Eastern Ethiopia, 2021. Case-control study. Front Pead, 2022. 9. 10.3389/fped.2021.795637 PMC8918646 35295317

[26] Ababa A. Capacity needs assessment for nutrition monitoring, evaluation and policy research in Ethiopia. 2020.

[27] Bach A (2020). Multisectoral integration of nutrition, health, and agriculture: implementation lessons from Ethiopia. FoodNutr Bull.

[28] Bachewe FN, Minten B. The rising costs of nutritious foods: the case of Ethiopia. Volume 134. Intl Food Policy Res Inst; 2019.

[29] Exchange GFD. Dashboard: Country Fortification. Accessed 17Nov2019 from https://fortificationdata.org/country-fortification-dashboard, 2018.

[30] Ethiopia F (2016). National nutrition program 2016–2020.

[31] Ethiopia FDRo. The Seqota Declaration: Innovation Phase Investment Plan (2017–2020). Federal Democratic Republic of Ethiopia Addis Ababa, Ethiopia; 2018.

[32] Edris Y (2020). Neural tube defects and Associated factors among neonates admitted to the neonatal intensive care units in Hiwot Fana Specialized University Hospital, Harar, Ethiopia. Global Pediatr Health.

[33] Tefera M et al. Women’s Birth Experience and Neonatal Outcomes, Study Design and Methodology with Baseline Characteristics. A Hospital-Based Maternal Follow-up Study. 2021.

[34] Nigussie S (2022). Treatment outcome and associated factors among patients admitted with acute poisoning in a tertiary hospital in Eastern Ethiopia: a cross-sectional study. SAGE Open Medicine.

[35] Eyeberu A (2021). Neonatal mortality among neonates admitted to NICU of Hiwot Fana specialized university hospital, eastern Ethiopia, 2020: a cross-sectional study design. BMC Pediatr.

[36] Ibrahim AM (2021). The Effect of Admission Hypothermia for neonatal death among Neonates admitted to neonatal intensive care unit at Sheik Hassan Yabare Jigjiga University Referral Hospital in Jigjiga City, Somali Region, Eastern Ethiopia. Res Rep Neonatology.

[37] Anyanwu L-JC, Danborno B, Hamman WO (2015). The prevalence of neural tube defects in live born neonates in Kano, North-Western Nigeria. Sub-Saharan Afr J Med.

[38] Tadesse AW, Kassa AM, Aychiluhm SB. Determinants of Neural Tube Defects among Newborns in Amhara Region, Ethiopia: A Case-Control Study. International Journal of Pediatrics, 2020. 2020. 10.1155/2020/5635267 PMC7648700 33193764

[39] Tesfay FA, Aga FB, Teshome GS. Determinants of neural tube defect among children at zewditu memorial hospital, addis ababa, ethiopia a case control study. Int J Afr Nurs Sci, 2021: p. 100318.

[40] Atlaw D, Neural Tube Defect and Associated Factors in Bale Zone Hospitals, Ethiopia S, Preg Child J et al. Health 6: 412. doi: 10.4172/2376-127X. 1000412 Page 2 of 7 Volume 6• Issue 3• 1000412 through folic acid fortification worldwide [13]. Despite this fact, nearly two-thirds of Ethiopian women are victim of folic acid deficiency [14]. Worldwide, more than 300,000 babies are born with NTDs each year, serious birth defects of the brain and spine are a significant cause of infant death and lifelong disability [13]. Africa, it is said to affect approximately, 2019: p. 1–3.

[41] Zaheri F (2017). Risk factors associated with neural tube defects in infants referred to western iranian obstetrical centers; 2013–2014. Electron Physician.

[42] Carmichael S (2012). Higher diet quality reduces risks of neural tube defects and orofacial clefts. Arch Pediatr Adolesc Med.

[43] Boyles AL (2006). Neural tube defects and folate pathway genes: family-based association tests of gene–gene and gene–environment interactions. Environ Health Perspect.

[44] Bourouba R, Houcher B, Akar N (2018). Risk factors of neural tube defects: a reality of Batna region in Algeria. Egypt J Med Hum Genet.

[45] Greene ND, Copp AJ (2014). Neural tube defects. Annu Rev Neurosci.

[46] De Marco P (2011). Maternal periconceptional factors affect the risk of spina bifida-affected pregnancies: an italian case–control study. Child’s Nerv Syst.

[47] Giannelli M (2003). The effect of caffeine consumption and nausea on the risk of miscarriage. Paediatr Perinat Epidemiol.

[48] Weng X, Odouli R, Li DK (2008). Maternal caffeine consumption during pregnancy and the risk of miscarriage: a prospective cohort study. Am J Obstet Gynecol.

[49] Schmidt RJ (2009). Maternal caffeine consumption and risk of neural tube defects. Birth Defects Res A Clin Mol Teratol.

[50] Lee H, Nagele RG, Pietrolungo JF (1982). Toxic and teratologic effects of caffeine on explanted early chick embryos. Teratology.

